# Association Between What People Learned About COVID-19 Using Web Searches and Their Behavior Toward Public Health Guidelines: Empirical Infodemiology Study

**DOI:** 10.2196/28975

**Published:** 2021-09-02

**Authors:** Ikpe Justice Akpan, Obianuju Genevieve Aguolu, Yawo Mamoua Kobara, Rouzbeh Razavi, Asuama A Akpan, Murali Shanker

**Affiliations:** 1 Department of Management & Information Systems Kent State University New Philadelphia, OH United States; 2 Infectious Disease Internal Medicine Department Yale School of Medicine Yale University New Haven, CT United States; 3 Statistical and Actuarial Sciences Western University London, ON Canada; 4 Department of Management & Information Systems Kent State University Kent, OH United States; 5 Research and Development Ibom International Center for Research and Scholarship Windsor, ON Canada

**Keywords:** internet, novel coronavirus, SARS-CoV-2, COVID-19, infodemiology, misinformation, conspiracy theories, public health

## Abstract

**Background:**

The use of the internet and web-based platforms to obtain public health information and manage health-related issues has become widespread in this digital age. The practice is so pervasive that the first reaction to obtaining health information is to “Google it.” As SARS-CoV-2 broke out in Wuhan, China, in December 2019 and quickly spread worldwide, people flocked to the internet to learn about the novel coronavirus and the disease, COVID-19. Lagging responses by governments and public health agencies to prioritize the dissemination of information about the coronavirus outbreak through the internet and the World Wide Web and to build trust gave room for others to quickly populate social media, online blogs, news outlets, and websites with misinformation and conspiracy theories about the COVID-19 pandemic, resulting in people’s deviant behaviors toward public health safety measures.

**Objective:**

The goals of this study were to determine what people learned about the COVID-19 pandemic through web searches, examine any association between what people learned about COVID-19 and behavior toward public health guidelines, and analyze the impact of misinformation and conspiracy theories about the COVID-19 pandemic on people’s behavior toward public health measures.

**Methods:**

This infodemiology study used Google Trends’ worldwide search index, covering the first 6 months after the SARS-CoV-2 outbreak (January 1 to June 30, 2020) when the public scrambled for information about the pandemic. Data analysis employed statistical trends, correlation and regression, principal component analysis (PCA), and predictive models.

**Results:**

The PCA identified two latent variables comprising past coronavirus epidemics (pastCoVepidemics: keywords that address previous epidemics) and the ongoing COVID-19 pandemic (presCoVpandemic: keywords that explain the ongoing pandemic). Both principal components were used significantly to learn about SARS-CoV-2 and COVID-19 and explained 88.78% of the variability. Three principal components fuelled misinformation about COVID-19: misinformation (keywords “biological weapon,” “virus hoax,” “common cold,” “COVID-19 hoax,” and “China virus”), conspiracy theory 1 (ConspTheory1; keyword “5G” or “@5G”), and conspiracy theory 2 (ConspTheory2; keyword “ingest bleach”). These principal components explained 84.85% of the variability. The principal components represent two measurements of public health safety guidelines—public health measures 1 (PubHealthMes1; keywords “social distancing,” “wash hands,” “isolation,” and “quarantine”) and public health measures 2 (PubHealthMes2; keyword “wear mask”)—which explained 84.7% of the variability. Based on the PCA results and the log-linear and predictive models, ConspTheory1 (keyword “@5G”) was identified as a predictor of people’s behavior toward public health measures (PubHealthMes2). Although correlations of misinformation (keywords “COVID-19,” “hoax,” “virus hoax,” “common cold,” and more) and ConspTheory2 (keyword “ingest bleach”) with PubHealthMes1 (keywords “social distancing,” “hand wash,” “isolation,” and more) were *r*=0.83 and *r*=–0.11, respectively, neither was statistically significant (*P*=.27 and *P*=.13, respectively).

**Conclusions:**

Several studies focused on the impacts of social media and related platforms on the spreading of misinformation and conspiracy theories. This study provides the first empirical evidence to the mainly anecdotal discourse on the use of web searches to learn about SARS-CoV-2 and COVID-19.

## Introduction

### Overview

A novel coronavirus initially named 2019-nCoV emerged in Wuhan, China, and was formally reported to the World Health Organization (WHO) on December 31, 2019 [1-3]. Further scientific evidence soon unveiled the semblance of the 2019-nCoV’s genome sequence to a previous epidemic, the severe acute respiratory syndrome (SARS), a disease epidemic caused by SARS-CoV, which broke out in Foshan, China, in 2002 [4,5]. Some initial studies also identified similar features that were related to the Middle East respiratory syndrome (MERS) epidemic caused by MERS-CoV as the causative agent [6].

The outbreak was originally named 2019-nCoV on January 13, 2020, the same day that the first imported case outside China occurred in the Philippines and other countries [7]. The spread of 2019-nCoV continued across many countries, causing the WHO to declare the outbreak a pandemic [6]. The 2019-nCoV was later renamed SARS-CoV-2 and identified as the causative agent of COVID-19 in February 2020 [8,9]. The highly contagious COVID-19 spread rapidly globally and caught the world unprepared. With no adequately planned health communication strategies, panic ensued, while confirmed cases of infections and deaths from COVID-19 increased rapidly worldwide [3]. The public rushed to internet platforms to learn about the outbreak through Google searches, online news outlets, and social media platforms [10-15].

In March 2020, the WHO launched a free online introductory training course in different languages, including English, French, Spanish, and Chinese, to make the public aware of the contagious COVID-19 [6]. However, it is unclear how many people knew about or used the free training lessons about COVID-19 that the WHO had made available via its website [6]. Instead, several studies suggest that the public flocked to the internet to learn about SARS-CoV-2 and COVID-19 through web searches, online news outlets, and social media [16,17]. Analyzing how people search and navigate the World Wide Web and other internet platforms for health-related information can provide valuable insights into the health-related behavior of populations [18-20]. The public’s preference for online health information closely matches the field of infodemiology, a term that is a portmanteau of information and epidemiology. According to Eysenbach [18], the term is defined as the science of distribution and determinants of information in an electronic medium—specifically the internet—or in a population, with the aim to inform public health and public policy. Considering the global spread of COVID-19, using the internet to learn or gain information about the pandemic in this digital age is not surprising, as internet use has become pervasive worldwide [21,22]. Several studies have examined social media’s influence on what people learned and the appropriate behaviors toward misinformation and conspiracy theories [14,15]. Similarly, Sulyok et al [12] examined the impact of web searches on the confirmed cases of COVID-19 in Europe, while Neely et al [23] investigated information-seeking behaviors on social media regarding the pandemic.

Miller [14,15] identified political leaders’ failure in sensitizing the public as a motivating factor that pushed people to the internet as an alternative information source to learn about the COVID-19 pandemic. Misinformation had started flooding the web right from the initial stage of the emergence of the novel coronavirus, mainly from user-created content on social media [24]. Thus, as people turned to the web to search for information, there was limited nontechnical information for the nonexpert public about the coronavirus. Rather, people either got exposed to learning incorrect information about SARS-CoV-2 and COVID-19 or embraced fake news, misinformation, and conspiracy theories, with grave consequences [16]. Some of the unfounded misinformation included misconstruing COVID-19 as a “common cold” or as a hoax, which made people have a false sense of immunity, while others ignored any public health safety measures [11]. Similarly, the conspiracy theories propagated online included that COVID-19 was a bioweapon, that China intentionally released the virus to reduce the world population, and that 5G technology contributed to the fast spread of the pandemic. These beliefs initially led to the hoarding of essential goods as well as racial attacks against Chinese and other Asian people [14,15,25,26].

Other studies examined the role of social media and internet news outlets in generating misinformation, disinformation, fake news, and conspiracy theories about COVID-19 [25,27-29]. These studies tend to leave out the aspect of web searching, such as the use of Google searches, which constitutes a major channel through which the public obtain health-related information [30,31].

### Research Objectives

This paper undertakes the first empirical investigation using a web search to learn about SARS-CoV-2 and COVID-19 and people’s attitudes toward public health guidelines as expressed in the following research objectives:

1. Determine what people learned about the COVID-19 pandemic through web searching.
2. Examine any association between what people learned about COVID-19 and behavior toward public health guidelines.
3. Analyze the impact of misinformation and conspiracy theories about the COVID-19 pandemic on people’s behavior toward public health measures.

These objectives are developed into research hypotheses in the sections that follow.

### Theoretical Background

#### The Connectivism Learning Theory

This section examines the connectivism learning theory, which explains the use of digital platforms to enable learning [32,33]. This study employs this approach to explore how people learned about SARS-CoV-2 and COVID-19 through web searching and the potential behavioral implications toward public health guidelines, which scientists and medical experts recommend as ways to check the spread of COVID-19. For example, the study investigates if learning through web searching helped people acquire accurate knowledge or misinformation and conspiracy theories about the COVID-19 pandemic and its implications. Also, recent studies show that many people have yet to understand the science and the concept of SARS-CoV-2 and the disease, COVID-19, which increases the danger of embracing misinformation [14,28]. Several web platforms, including social media, online news, and other internet channels, contribute significantly to misinformation and conspiracy theories [14,34].

According to Dunaway [33], the connectivism theory developed by George Siemens analyzes the use of digital devices, computer networks, and electronic platforms to learn. The view is considered a pedagogical strategy for the digital age, emphasizing knowledge sharing across an interconnected web and internet network [32,33]. The approach focuses on knowledge acquisition using information technology platforms and learning from multiple sources, developing skills, and disseminating information [32]. The platforms incorporate information on social media, internet websites or blogs, and search engines that users can employ to learn and exchange knowledge, skills, and expertise [28,33].

#### Implications of the Connectivism Learning Theory

One of the implications of the connectivism learning theory is that learning can occur outside the traditional classroom by using networked systems that enhance connections, interactions, and collaborations among learners [29]. However, some learning theory experts criticize the connectivism theory for not offering any improvement to the actual learning method other than using Web 2.0 and related platforms [32,33]. Hence, it cannot be deemed a substantive learning theory. Instead, it provides a bridge to other pedagogical methods: behaviorism, cognitivism, and constructivism. The core of the Siemens and Downes connectivism idea aims to move away from the traditional classroom learning techniques to a new theory of learning that embraces technology as the learning tool, which can inspire the new generation of learners and educators [32,33]. Thus, the theory draws its strength from web-based activities [29].

The key benefit of the method is its intuitiveness and its ability to captivate learners due to the ubiquitous use of the internet in today’s world. The following principles contribute to the popularity of connectivism as a learning theory [33]:

1. Learning and knowledge rest in diversity of opinions, as experienced today.
2. Learning is a process of connecting specialized nodes or information sources.
3. Learning may reside in nonhuman appliances.
4. The capacity to know more is more critical than what is currently known.
5. Nurturing and maintaining connections help to facilitate continual learning.
6. Ability to see connections between fields, ideas, and concepts is a core skill.
7. Currency (accurate, up-to-date knowledge) is the intent of all connectivism learning activities.
8. Decision making is itself a learning process. Choosing what to learn and the meaning of incoming information is seen through a shifting reality.

The connectivism learning theory, as explained above, closely mirrors the use of Google Trends and other internet platforms to learn about the outbreak of SARS-CoV-2 and COVID-19, especially where the masses did not get adequate, timely information about the coronavirus from the public agencies [35,36].

The connectivism learning theory is well suited to personal study and self-regulated learning [37,38]; in this case, how individual members of the public learned about SARS-CoV-2 and COVID-19 using web searches in the first 6 months of the COVID-19 pandemic.

### An Overview of the SARS-CoV-2 Outbreak and the COVID-19 Pandemic

#### The Global Impacts of the SARS-CoV-2 Outbreak

The COVID-19 pandemic has caused severe problems ranging from health crises to psychological, social, business, and economic consequences all over the world [16,18,39]. Meanwhile, there is currently no specific cure for COVID-19. However, there has been significant progress in technological advances leading to substantial breakthroughs in vaccine discovery and development through the pioneering efforts of Pfizer, Moderna, and other companies from the United Kingdom, India, China, and other countries [40]. Administering the COVID-19 vaccines is ongoing worldwide, while several other vaccine discoveries and developments are in progress [41]. In the meantime, ongoing prevention, monitoring, and public health awareness are essential to mitigate the public health and economic burdens. The most important prevention strategy is to understand the disease and how it spreads.

#### Transmission

Coronavirus transmission primarily occurs through respiratory droplets released from infected persons during coughing, sneezing, or speech. One can also become infected with the virus via contact with contaminated surfaces. The virus can remain infectious in the air for 3 hours and on inanimate surfaces for up to 9 days or longer. This has implications for nosocomial spread and superspreading events [42]. The virus has also been isolated from blood, urine, and stool specimens. It is important to note that asymptomatic infected people may not be aware that they are infected because they do not have the symptoms or may not recognize the symptoms. Infected individuals can be contagious for up to 4 weeks and can unknowingly be spreading the infection during this time [42].

#### Clinical Presentation and Diagnosis

Symptoms usually appear 2 to 14 days after exposure. Most confirmed cases of SARS-CoV-2 infection are asymptomatic, and they recover without treatment. Common symptoms include fever, cough, shortness of breath, chills, myalgia, headache, sore throat, anosmia, and dysgeusia. Severe cases present with dyspnea, tachypnea, hypoxia (blood oxygen saturation ≤93%), a ratio of the arterial partial pressure of oxygen to the fraction of inspired oxygen of less than 300 mm Hg, and lung infiltration [43]. Some patients present with gastrointestinal symptoms, such as vomiting, diarrhea, and abdominal pain, as well as cardiovascular features, such as arrhythmia, shock, and acute cardiac injury [44]. There have been reports of asymptomatic carriers presenting with symptoms such as loss of smell and taste. In children, the majority present with mild (ie, fever, cough, fatigue, and congestion) or moderate (ie, pneumonia) symptoms [44]. Some may be asymptomatic. Children under 5 years old may present with respiratory organ failure.

Chest computed tomography scans shows a distinct appearance of ground-glass lung opacity, often bilateral, in patients who develop pneumonia [43]. Other radiographic features, such as “crazy-paving sign, multifocal organizing pneumonia, and architectural distortion in a peripheral distribution,” may appear with disease progression. Diagnostic testing is performed from respiratory (ie, nose, throat, and saliva) and serum samples, using a real-time reverse transcription–polymerase chain reaction panel** **or antibody test. Viral RNA has also been detected in stool and blood [5].

#### Complications

Some hospitalized patients develop thromboembolism, especially deep venous thrombosis, and pulmonary embolism. Other complications include microvascular thrombosis of the toes, clotting of catheters, myocardial injury with ST-segment elevation, and large vessel strokes. This complication may be associated with the release of high levels of inflammatory cytokines and activation of the coagulation pathway caused by hypoxia and systemic inflammation secondary to COVID-19 [45].

#### Prevention and Control

People must be well-informed. Infected persons must practice respiratory etiquette to avoid infecting others, including covering coughs and sneezes with a tissue and discarding it properly, coughing into the inside of the elbow, and covering the nose and mouth properly with a surgical face mask. Best practices include proper handwashing with soap and water for at least 20 seconds or at least 60% alcohol–based hand rub. Touched surfaces must be cleaned frequently with disinfectants. People must avoid touching the eyes, nose, and mouth with unwashed hands and they must avoid close contact with people who are ill [46]. The US Centers for Disease Control and Prevention (CDC) recommends that infected and exposed individuals must isolate or quarantine themselves, respectively, for at least 14 days. The CDC also recommends social distancing, including avoiding mass gatherings or large community events, shaking hands, or giving “high fives” [41]. In health care settings, standard contact and airborne precautions, as well as eye protection, should be used to mitigate the spread of SARS-CoV-2 [46]. There is no specific cure for COVID-19. Management is mainly supportive care and treatment of secondary infections. Severely ill patients may need advanced organ support.

## Methods

### Google Trends and Search Keywords About SARS-CoV-2 and COVID-19

This study used an infodemiology approach to evaluate the use of web searching to learn about SARS-CoV-2 and COVID-19. As an area of scientific research, infodemiology is a method or technique designed to measure and track health information “demand” automatically (eg, by analyzing search queries) as well as “supply” on the internet [18,20,47]. The goal is to inform public health policy and practice. This study uses data from Google Trends, a freely available online resource that provides information on what was and is trending based on actual users’ Google queries [11,48,49].

Google Trends offers various search options, such as “Trending Searches” (ie, trending queries for daily search trends and real-time searches in a selected region) or “Year in Search” (ie, what was trending in a specific area in a particular year). Another option is to “Explore,” which allows an investigation of an area of interest based on keywords over the selected periods and regions. This study uses the “Explore” option, which allowed data to be retrieved directly from the Google Trends “Explore” page in comma-separated values format. It is also important to note that Google Trends data points are normalized to have a maximum value of 100 and a minimum value of 0. We normalized the data set by dividing each data point by total searches of the geography and the time range it represents to compare relative popularity. Note that the value 0 does not necessarily indicate no searches but represents a significantly low search volume that does not warrant inclusion in the results [47].

In this study, we captured the worldwide Google Trends data covering the initial months of the SARS-CoV-2 outbreak from January 1 to June 30, 2020 (ie, 182 daily data points for each search term). Regarding the search terms, this paper employed 25 keywords and phrases used by the public to learn about the COVID-19 pandemic through web searches. We identified the search keywords (Table 1) through a literature survey of published documents indexed on the Web of Science. Six search terms were related directly to the ongoing pandemic: “nCoV,” “2019-nCoV,” “SARS-CoV-2,” “COVID-19,” “pandemic,” and “coronavirus.” Another six keywords addressed previous viral or coronavirus epidemics: “SARS-CoV,” “SARS,” “MERS-CoV,” “MERS,” “virus,” and “influenza.” The third category of search terms represented public health safety measures that experts recommended as guidelines to limit the spread of COVID-19: “social distancing,” “wear a facial mask,” and “wash hands.” The final category of keywords represented misinformation and conspiracy theories, such as “China virus,” “common cold,” and “bioweapon” (Table 1 [2,3,6,7,9,11,14,15,25-27,42,46,50-60]).

**Table 1:** Web search terms used to learn about SARS-CoV-2 and the COVID-19 pandemic, misinformation and conspiracy theories, and public health safety guidelines, based on a literature review.

Category	Search terms
COVID-19 and related epidemics	“2019-nCoV” [3], “nCoV” [3,9], “SARS-CoV-2” [42], “COVID-19” [9,25,50], “pandemic” [6,7], “MERS-CoV” [51,52,60], “MERS^a^” [51,53], “SARS-CoV” [42,52], “SARS^b^” [52,53], “virus” [54], “coronavirus” [2,53,55], “influenza” [46], and “flu” [59]
Misinformation and conspiracy theories	“virus hoax” [14,15], “injecting or ingesting bleach” [56], “5G” or “@5G” technology enhancing the spread of the virus [25,27], “COVID-19 hoax” [14,15,26], “common cold” (“commoncold2020”) [11], “China virus” [50], and “bioweapons” created by China [14,25,60]
Public health measures	“social distancing” [57], “wash hands” or “hands wash” [57], “wear a facial mask” [58], “isolation” [57], and “quarantine” [57]

^a^MERS: Middle East respiratory syndrome.

^b^SARS: severe acute respiratory syndrome.

### Research Hypotheses

#### Hypothesis 1

For the purpose of determining what people learned about the COVID-19 pandemic through web searching, we defined the null and alternative hypotheses as follows:

- Hypothesis 1_0_: People did not learn about COVID-19 through web searching using the identified keywords.
- Hypothesis 1_1_: People learned about COVID-19 through web searching using the identified keywords.

#### Hypothesis 2

Based on the literature, using a web search to learn about a subject of interest can influence the learner’s decision making and actions [61]. On this premise, this study examined any association between what people learned about COVID-19 and people’s behavior toward the public health guidelines. We developed two separate hypotheses (Hypotheses 2 and 3). The first aspect related to web searching to learn about COVID-19 (ie, concept, science, and structure of SARS-CoV-2 and COVID-19), while the second aspect evaluated learning about misinformation and conspiracy theories as well as the behavioral response to the public health measures. The null and alternative hypotheses regarding the first aspect are as follows:

- Hypothesis 2_0_: There is no association between what people learned about COVID-19 through web searching and behavior toward public health measures.
- Hypothesis 2_1_: There is an association between what people learned about COVID-19 through web searching and behavior toward public health measures.

#### Hypothesis 3

There is a widely held assertion that misinformation and conspiracy theories about the COVID-19 pandemic have had a significant impact on people’s behavior toward public health measures. We defined the null and alternative hypotheses for learning about misinformation as follows:

- Hypothesis 3A_0_: There is no association between misinformation learned about COVID-19 and people’s behavior toward public health measures.
- Hypothesis 3A_1_: There is an association between misinformation learned about COVID-19 and people’s behavior toward public health measures.

Similarly, we defined the null and alternative hypotheses for learning about conspiracy theories as follows:

- Hypothesis 3B_0_: There is no association between conspiracy theories learned about COVID-19 and people’s behavior toward public health measures.
- Hypothesis 3B_0_: There is an association between conspiracy theories learned about COVID-19 and people’s behavior toward public health measures.

### Data Analysis

Data analysis employed statistical trends and graphical visualization, correlation and regression, principal component analysis (PCA), and predictive models [12,56,62-64]. The statistical trends and analyses involved evaluating relationships among the listed variables using the statistical trends, including graphical display, correlation, and PCA, which helped determine the predictiveness of the learning attributes and learners’ actions toward public health guidelines. We used the JMP 15 package from SAS software (SAS Institute Inc) [65] for statistical analysis and Microsoft Excel 2019 to create the charts and graphs. We also used SPSS software (version 27; IBM Corp) to compute the correlation matrix and the PCA as well as statistical packages in R (version 4.0.5; The R Foundation) for the linear modeling. The evaluation helped establish the correlation between the study attributes.

## Results

### Overview

The data analyzed in this study came from the Google Trends worldwide index covering the period from the initial outbreak of COVID-19 on January 1, 2020, up to June 30, 2020, when the pandemic became widely known [2]. The outbreak had been reported to the WHO’s office in China on December 31, 2019 [6]. The reason for focusing on the first 6 months of the pandemic was to capture what people learned during the early days of the outbreak as well as the possible impacts of what people learned through web searching on individuals’ attitudes toward public health safety measures.

To better understand the characteristics of Google Trends data, we have presented the summary statistics of the daily search index for each of the 25 keywords or search terms and phrases (Table S1 in Multimedia Appendix 1). The average normalized scores for the terms varied from 2.65 (“ingesting bleach”) to 39.75 (“SARS-CoV-2”), as shown in Table S1 in Multimedia Appendix 1.

### Temporal Trends: Using Web Searches to Learn About SARS-CoV-2

The keywords employed to conduct web searches indicate what people learned about the COVID-19 pandemic [66]. As presented in Table 1, some of the search keywords addressed the novel coronavirus directly, while others examined misinformation and conspiracy theories.

**Figure 1:**
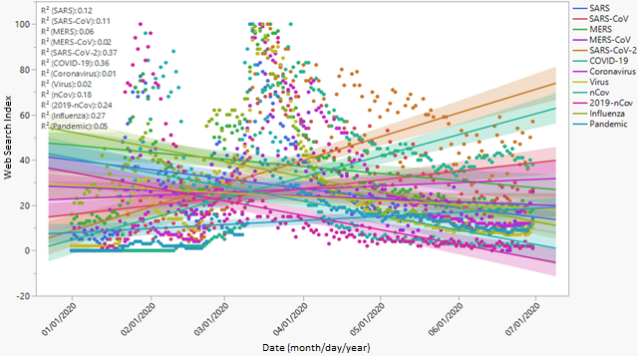
The keywords used by people to learn about SARS-CoV-2 and COVID-19 through web searches. MERS: Middle East respiratory syndrome; SARS: severe acute respiratory syndrome.

Figure 1 presents the first category of web search terms that people used to learn about the COVID-19 pandemic. In the early period, most people used keywords and phrases that explain previous coronavirus epidemics, including “influenza,” “MERS,” “MERS-CoV,” “SARS,” and “virus.” Although scientists ruled out the past epidemics, the WHO officials highlighted those terms as examples of past coronavirus outbreaks during press briefings [6]. The use of those keywords in the web searches nosedived after the WHO formally named the novel coronavirus and the disease (ie, “nCoV,” “2019-nCoV,” “SARS-CoV-2,” and “COVID-19”). The coefficients of determination (*R*^2^) of the keywords are 0.37 for “SARS-CoV-2,” 0.36 for “COVID-19,” 0.27 for “influenza,” 0.24 for “2019-nCoV,” 0.18 for “nCoV,” 0.12 for “SARS,” and 0.11 for “SARS-CoV,” indicating the proportion of the variation in the search index over the period for the listed keywords. Similarly, the search terms “MERS,” “MERS-CoV,” “pandemic,” “virus,” “coronavirus,” “nCoV,” and “2019-nCoV” had *R*^2^ values of less than 0.1.

**Figure 2:**
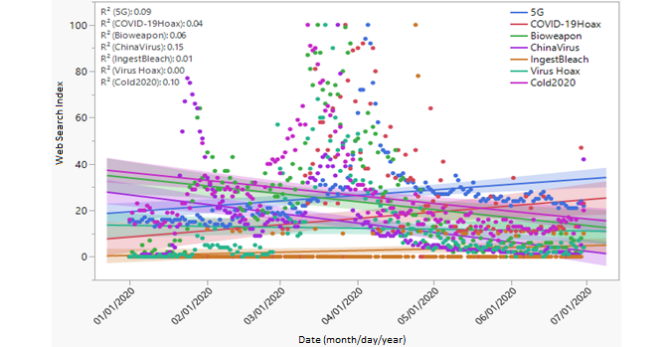
Worldwide search index showing the learning terms that represent misinformation and conspiracy theories about COVID-19.

The second category of keywords involved misinformation and conspiracy theories (Figure 2). The variation in the use of the terms was measured using coefficients of determination (*R*^2^), which were 0.15 for “China virus,” 0.10 for “common cold,” and 0.09 for “5G” or “@5G.” Most searches in the initial months of the outbreak used the keywords “common cold” (“cold2020”), “biological weapon,” and “China virus,” thus encouraging the misconception about SARS-CoV-2 as a “common cold,” a “biological weapon,” or a “China virus” [50]. Some studies explain that the purpose of releasing the coronavirus was to reduce the world population [14,25]. However, web searches using these terms fell continuously over time to a near-zero search index, while new words (“5G” and “COVID-19 hoax”) surfaced and increased significantly (Figure 2).

**Figure 3:**
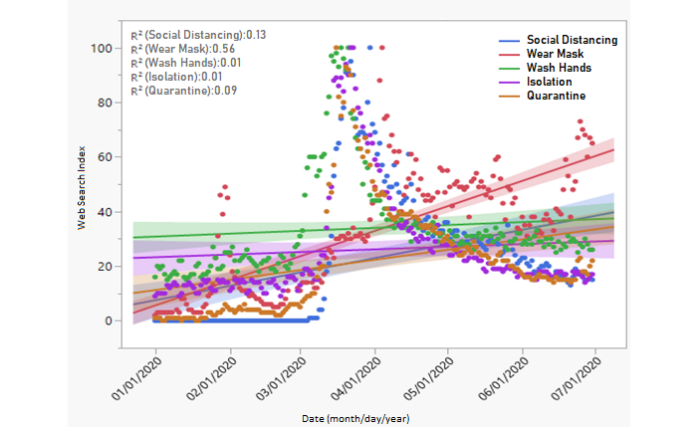
Trend analysis showing the web search index for learning about public health safety measures.

The third segment of the trend analysis involved web searches to learn about public health measures (Figure 3). The results show that there was little or no interest in learning about wearing a facial mask (“wear mask”) and maintaining social distancing (“social distancing”) at the start of the pandemic. But the trends changed quite quickly, recording a dramatic increase from a search index of 0 at the beginning of the outbreak to achieving a maximum search index of 100 in March and April 2020, as the pandemic spread worldwide. The coefficients of determination were as follows: “wear mask” (*R*^2^=0.56), “social distancing” (*R*^2^=0.13), and “quarantine” (*R*^2^=0.09). The increases, especially regarding “wear mask” and “social distancing,” were sustained for a long time.

### What Did People Learn About COVID-19 Through Web Searches?

**Figure 4:**
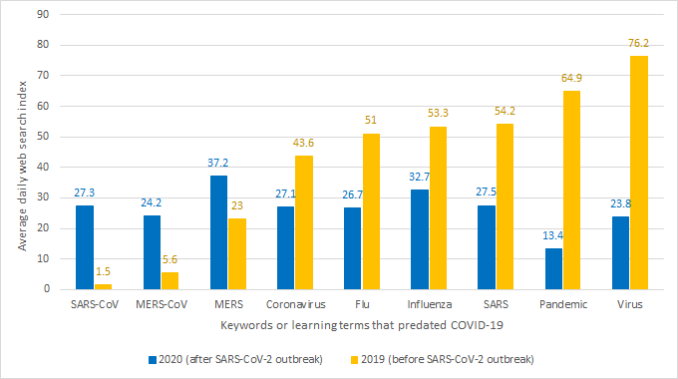
The average search index for pre-existing keywords before and during the COVID-19 pandemic. MERS: Middle East respiratory syndrome; SARS: severe acute respiratory syndrome.

The keywords identified above approximate what people learned about SARS-CoV-2 and COVID-19 through web searches. However, some of the search keywords existed before the ongoing pandemic, while some terms referred to previous coronavirus epidemics (eg, “SARS,” “SARS-CoV,” “MERS,” “MERS-CoV,” “influenza,” “virus,” “pandemic,” and “coronavirus”). It is plausible to argue that the search index for the pre-existing keywords listed represent purposes other than learning about COVID-19. Based on this assumption, we conducted a dependent two-sample *t* test to examine the difference in the mean search index of the pre-existing keywords in the previous years before the COVID-19 outbreak and during the ongoing pandemic. Figure 4 compares the mean search index before and after the outbreak for each keyword.

The null hypothesis was that the search indexes of each learning term—“SARS,” “SARS-CoV,” “MERS,” “MERS-CoV,” “influenza,” “virus,” “pandemic,” and “coronavirus”—before and after the outbreak of COVID-19 would be equal, and the alternative hypothesis was that they would be unequal. The results, as seen in Figure 4, show that the differences in the mean search indexes before and during the ongoing pandemic were more than 60% in all cases. Also, the P values were close to zero for all the variables. We rejected the null hypothesis and concluded that the significant differences in the mean search indexes of the variables were due to the ongoing COVID-19 pandemic. Unexpectedly, the mean search index for some pre-existing keywords (eg, “flu,” “influenza,” “SARS,” “pandemic,” and “virus”) declined during the pandemic. 

### What Search Terms Contributed to Learning About SARS-CoV-2 and COVID-19?

**Figure 5:**
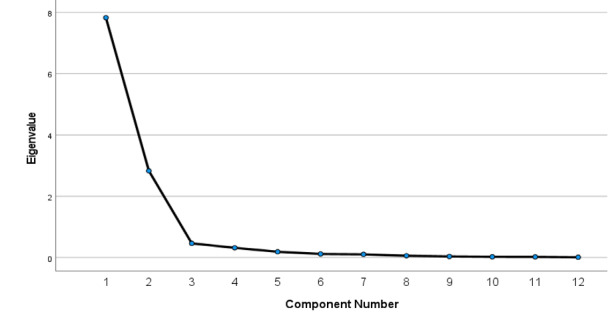
Scree plot of COVID-19 learning terms; two principal components extracted 88.78% of the total variation.

PCA was employed to evaluate the underlying latent variable of the search terms that contributed to learning about COVID-19. Based on the scree plot and the elbow rule, we can limit the factors extracted to the first two principal components (Figure 5): the keywords that address previous epidemics (pastCoVepidemics) and the keywords that explain the ongoing pandemic (presCoVpandemic). A scree plot is a graphical representation of the percentage variability explained by each principal component.

The first two underlying components explained a total of 88.78% of the variation in learning terms, with the first (pastCoVepidemics) and second (presCoVpandemic) components determining about 65.2% and 23.58%, respectively, of the information about COVID-19 from the 12 search keywords (Table 2).

**Table 2:** Total explained variance for search keywords used to learn about COVID-19.

Measure	PC^a^ 1	PC 2	PC 3	PC 4	PC 5	PC 6	PC 7	PC 8	PC 9	PC 10	PC 11	PC 12
Total initial eigenvalue	7.82	2.83	0.46	0.32	0.19	0.12	0.11	0.06	0.04	0.03	0.02	0.01
Variance, %	65.20	23.58	3.86	2.65	1.57	0.98	0.88	0.49	0.29	0.21	0.19	0.09
Cumulative variance, %	65.20	88.78	92.64	95.30	96.87	97.84	98.72	99.21	99.51	99.72	99.91	100

^a^PC: principal component.

Linear combinations of the two components (pastCoVepidemics and presCoVpandemic) are as follows:

- PastCoVepidemics = 0.98 “virus” + 0.934 “coronavirus” + 0.929 “MERS” + 0.923 “flu” + 0.858 “MERS-CoV” + 0.858 “SARS” + 0.791 “SARS-CoV” + 0.799 “pandemic” + 0.814 “influenza”
- PresCoVpandemic = –765 “nCoV” + 0.784 “COVID-19” + 0.766 “SARS-CoV-2.”

Table 3 shows the weights (loadings) of the terms for the two components. Note that we record loadings greater than 0.6 to combine only search keywords that have a high correlation with the component in the linear combinations.

**Table 3:** Component matrix and weight loadings for search keywords used to learn about COVID-19.

Component^a^	Weight loading for each keyword											
	Virus 2020	Coronavirus 2020	MERS^b^ 2020	Flu 2020	MERS-CoV 2020	SARS^c^ 2020	Influenza 2020	Pandemic 2020	SARS-CoV 2020	COVID-19	SARS-CoV-2	2019-nCoV
1	0.98	0.934	0.929	0.923	0.858	0.858	0.814	0.799	0.791	0.562	0.601	0.45
2	–0.059	0.278	–0.305	–0.278	–0.121	–0.443	–0.477	0.315	0.505	0.784	0.766	–0.765

^a^Component 1 is pastCoVepidemics (keywords that address previous epidemics) and component 2 is presCoVpandemic (keywords that explain the ongoing pandemic).

^b^MERS: Middle East respiratory syndrome.

^c^SARS: severe acute respiratory syndrome.

### What Terms Fueled Misinformation and Conspiracy Theories About COVID-19?

**Figure 6:**
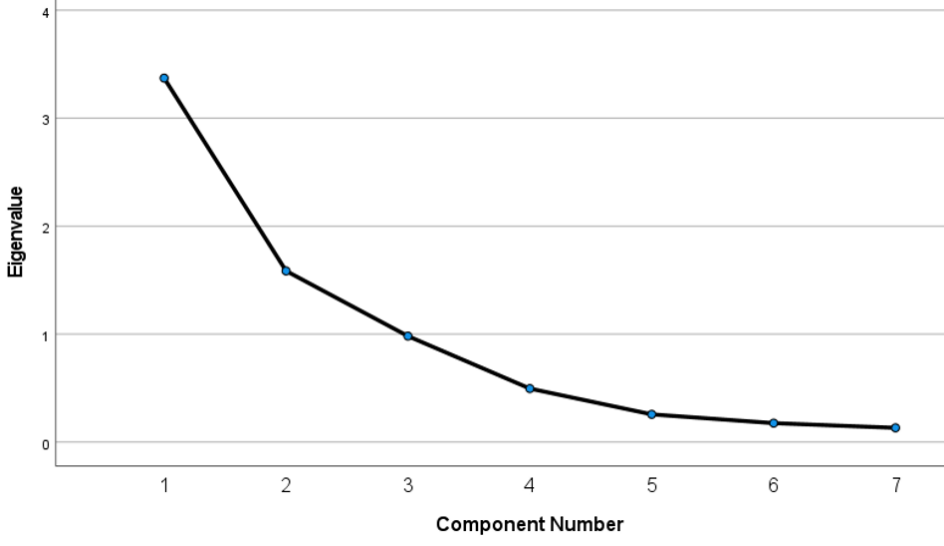
Scree plot of misinformation and conspiracy theory terms.

We identified eight search keywords from the literature that denote misinformation and conspiracy theories (Table 1). We also performed a PCA to evaluate the search terms that fueled misinformation and conspiracy theories. The results, as seen in Table 4 and Figure 6, identified three principal components and their variabilities based on the elbow rule: principal component 1 (48.17%), principal component 2 (22.65%), and principal component 3 (14.03%).

**Table 4:** Total variance explained involving terms that fueled misinformation and conspiracy theories.

Measure	PC^a^ 1	PC 2	PC 3	PC 4	PC 5	PC 6	PC 7
Total initial eigenvalue	3.372	1.586	0.982	0.496	0.257	0.176	0.132
Variance, %	48.171	22.652	14.026	7.085	3.669	2.509	1.888
Cumulative variance, %	48.171	70.823	84.849	91.934	95.603	98.112	100

^a^PC: principal component.

The three components explained 84.85% of the variation in the search keywords under misinformation and conspiracy theories. The first component represented misinformation. We quantified the daily number of misinformation terms searched using the linear combination as follows:

- Misinformation = 0.789 “common cold” + 0.928 “bioweapon” + 0.908 “virus hoax” + 0.875 “cold 2020” + 0.692 “COVID-19 hoax” + 0.60 “China virus.”

The second and third components addressed two conspiracy theories (ConspTheory1 and ConspTheory2), which speculated that 5G technology contributes to the spreading of COVID-19, that COVID-19 is a “China virus” that was intentionally created and released, and that ingesting or injecting bleach can cure COVID-19 infection or kill the virus. The results present these variables in the separate components (Table 5) as follows:

- ConspTheory1 = 0.786 “@5G”
- ConspTheory2 = 0.97 “ingest bleach.”

**Table 5:** Component matrix and weight loading for terms that fueled misinformation and conspiracy theories.

Component^a^	Weight loading for each keyword							
	Bioweapon 2020	Virus hoax	Common cold 2020	COVID-19 hoax	China virus	@5G	Ingest bleach	
1	0.928	0.908	0.789	0.692	0.601	0.471	–0.045	
2	–0.152	0.033	–0.438	0.624	–0.558	0.786	0.226	
3	0.03	0.005	0.093	–0.044	0.059	–0.145	0.973	

^a^Component 1 is misinformation and is not included, component 2 is the conspiracy theory that 5G technology contributes to the spreading of COVID-19, and component 3 is the conspiracy theory that ingesting or injecting bleach can cure COVID-19 infection or kill the virus.

### Public Health Safety Measures

**Figure 7:**
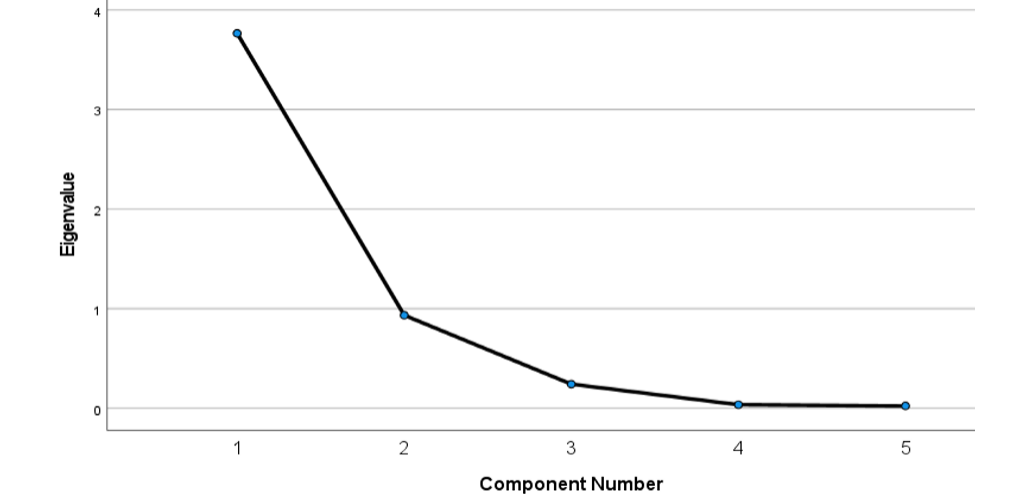
Scree plot of public health measures against COVID-19; two components extracted 93.99% of the variability in the search index.

This section investigates the impacts of what people learned through web searches on behaviors toward public health safety measures against COVID-19. Based on the elbow rule, the PCA identified two public health measure components, which we labeled as PubHealthMes1 and PubHealthMes2. The two components accounted for the variability in the search index of keywords used to learn about the public health measures against the spread of COVID-19 (Figure 7). The first and second components explained more than 75.4% and 18.7% of the variability, respectively; that is, the first two components explained about 94% of the variability (Table 6).

**Table 6:** Total explained variance for terms that explained the public health measures against COVID-19.

Measure	PC^a^ 1	PC 2	PC 3	PC 4	PC 5
Total initial eigenvalue	3.768	0.933	0.24	0.035	0.023
Variance, %	75.359	18.665	4.799	0.71	0.466
Cumulative variance, %	75.359	94.025	98.824	99.534	100

^a^PC: principal component.

The first component, PubHealthMes1, includes the keywords “social distancing,” “wash hands,” “isolation,” and “quarantine.” In the second component, PubHealthMes2, the keyword “wear mask” explained 84.7% of the variability (Table 7):

- PubHealthMes1 = 0.953 “social distancing” + 0.847 “wash hands” + 0.953 “isolation” + 0.99 “quarantine”
- PubHealthMes2 = 0.847 “wear mask.”

**Table 7:** Component matrix and weight loading for terms that explained the public health measures.

Component^a^	Weight loading for each keyword				
	Quarantine	Social distancing	Isolation	Wash hands	Wear mask
1	0.99	0.953	0.953	0.847	0.503
2	–0.028	0.143	–0.223	–0.38	0.847

^a^Component 1 is public health measures represented by the keywords “social distancing,” “wash hands,” “isolation,” and “quarantine,” and component 2 is the public health measure represented the keyword “wear mask.”

### Analysis of the Relationships Among the Principal Components

**Figure 8:**
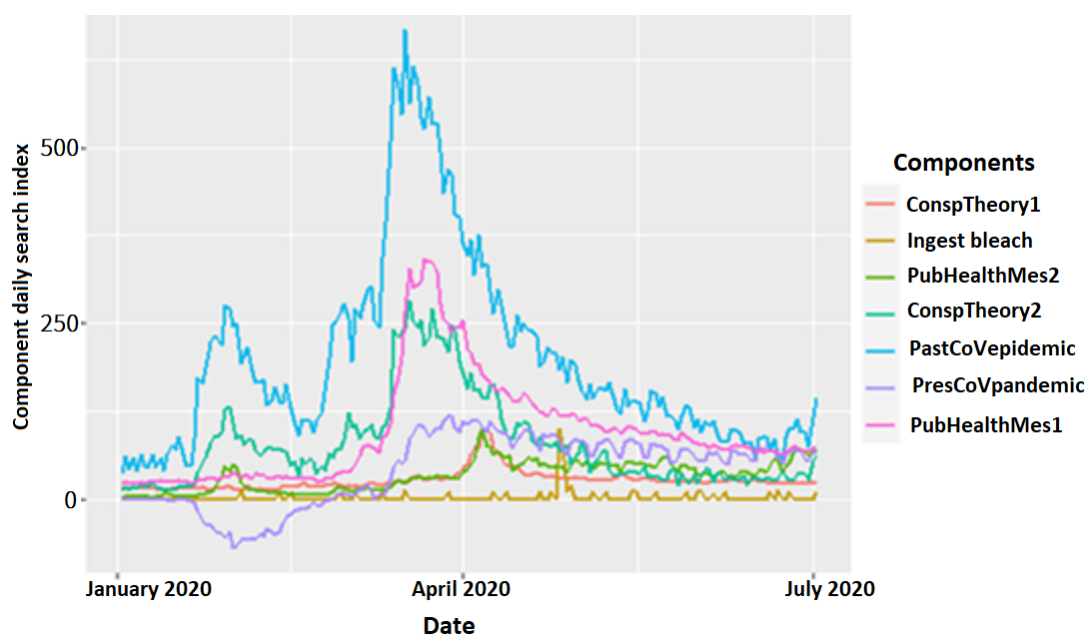
Daily search index of the principal components. ConspTheory1: conspiracy theory 1; ConspTheory2: conspiracy theory 2; pastCoVepidemics: keywords that address previous epidemics; presCoVpandemic: keywords that explain the ongoing pandemic; PubHealthMes1: public health measures 1; PubHealthMes2: public health measures 2.

This section presents further analysis that tested the hypothesis raised in the earlier section using built predictive models. The variables identified the linear combination of search keywords significantly correlated (loading >0.6) to the principal components discussed in the Results subsections above. Also, Tables 2 to 7 present the underlying latent variables of the 25 search terms used to learn about COVID-19, the misinformation and conspiracy theories, and the public health measures. As stated, Figure 8 shows the underlying variables.

Here, we examine how the underlying variables and the search terms impacted learning and behavior toward the public health measures: learning about COVID-19 (pastCoVepidemics and presCoVpandemic), misinformation and unproven or misleading assertions (misinformation), conspiracy theories (ConspTheory1 and ConspTheory2), and public health safety measures (PubHealthMes1 and PubHealthMes2).

The results show three essential highlights from the daily search index. First, the most popular search terms used at the initial outbreak of the pandemic in early January 2020 were terms representing misinformation and past epidemics. The search keywords that represented conspiracy theories were not used until May 2020. Also, the use of learning terms that directly explained COVID-19 (ie, presCoVpandemic) corresponded with the WHO’s naming and renaming of the coronavirus and the disease (ie, “2019-nCoV,” “SARS-CoV-2,” and “COVID-19”) in January, February, and March 2020.

### Information Learned Versus Behavior Toward Public Health Measures

**Figure 9:**
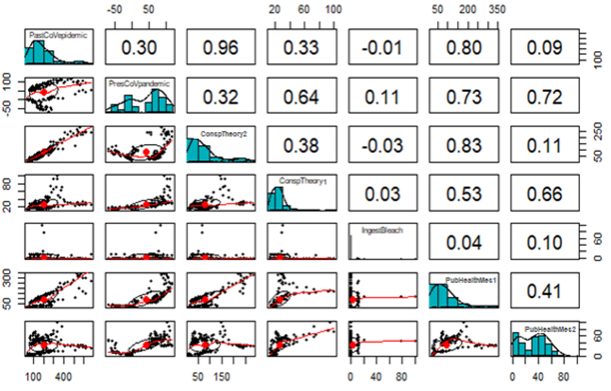
Panel pair plots of linear correlations between misinformation and conspiracy theory keywords. pastCoVepidemics: keywords that address previous epidemics; presCoVpandemic: keywords that explain the ongoing pandemic; PubHealthMes1: public health measure 1; PubHealthMes2: public health measure 2.

We employed correlation analysis among the variables, scatterplots, and their histograms to examine the relationship between what people learned and their attitudes toward public health measures (Figure 9).

PubHealthMes1 has a robust positive relationship with pastCoVepidemics (*r*=0.80) and a moderate positive relationship with presCoVpandemic (*r*=0.73), which implies the effectiveness of learning keywords associated with past coronavirus epidemics (eg, “SARS,” “SARS-CoV,” “MERS,” “MERS-CoV,” and more) and the ongoing pandemic (eg, “2019-nCoV,” “SARS-CoV-2,” and “COVID-19”). Similarly, PubHealthMes2 (ie, wearing a facial mask) has a moderate positive relationship with presCoVpandemic (*r*=0.71). There is a strong association between actions taken and the information learned. Figure 9 shows a correlation matrix.

**Figure 10:**
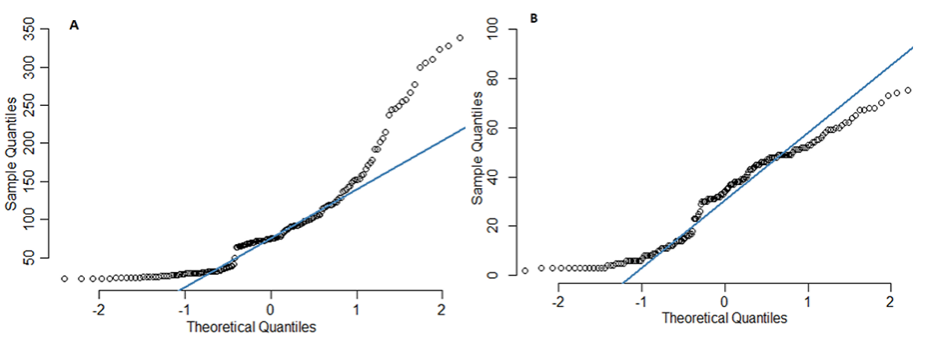
Quantile-quantile (Q-Q) plots of (A) PubHealthMes1 (public health measures 1, represented by the keywords “social distancing,” wash hands,” “isolation,” and “quarantine”) and (B) PubHealthMes2 (public health measures 2, represented the keyword “wear mask”).

Given such a strong linear relationship between the search terms and people’s behaviors and actions, a multiple linear regression model seems acceptable as a predictive model. But the data failed the assumption of normality, as shown by the quantile-quantile (Q-Q) plots in Figure 10, A and B. A normal Q-Q plot helps to compare two probability distributions, by plotting the residuals against theoretical quantiles. Plots A and B in Figure 10 show that most residuals are not lying on the diagonal line; hence, the data are not normally distributed.

From Figure 9, we can observe that the PubHealthMes1 histogram suggests a right-skewed distribution, while the PubHealthMes2 histogram suggests a bimodal distribution. To describe and explain the relationship between the public health measures and the terms that contributed to learning about COVID-19, misinformation, and conspiracy theories, we used the log-linear predictive model, since the data are based on a number of occurrences or frequency and not normally distributed. Also, the log-linear model does not need to satisfy any assumptions, which we represent as follows:



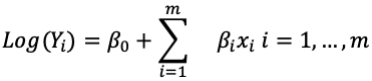



where *x_i_* is the covariate and *β_i_* is the parameter to be estimated.

The data obtained through the PCA have been fitted to a log-linear regression model using RStudio tools from R (version 4.0.5; The R Foundation). The regression model obtained becomes the following:

Log(PubHealthMes1) = 3.33 + 0.0024 pastCoVepidemics + 0.011 presCoVpandemic

The *R*^2^ of the model is 0.93 and the P value is <.001. All the coefficients in this model are significant at an α level of 0. From the model, we see that there is a 1.10 percentage (100 × [exp^0.011^ – 1]%) increase in the public health measure for every 1% increase in presCoVpandemic (ie, “2019-nCoV,” “SARS-CoV-2,” and “COVID-19”). This may be explained by links contained in recent COVID-19 pandemic articles on public health measures. For every 1% increase in past epidemic searches, there is a 0.25% increase in the searches of public health measures. Similarly, the regression model for PubHealthMes2 obtained becomes the following:

Log(PubHealthMes2) = 0.01 × presCoVpandemic + 2.35

The *R*^2^ of the new model is 0.50 and the P value is <.001. The two models show that both PubHealthMes1 and PubHealthMes2 are significantly predictive using the presCoVpandemic keywords. It shows that learning about the present pandemic creates an incentive for people to learn about public health measures, which we can approximate to a desired intention to comply with the measures.

### Misinformation, Conspiracy Theories, and Public Health Measures

This section examines the relationship between conspiracy theories, misinformation, and public health measures using correlation and predictive analyses. As discussed earlier, we recategorized the search terms representing misinformation and conspiracy theories into two principal components. Hypothesis 3A, as defined in the Research Hypotheses section, focuses on misinformation and public health guidelines. At the same time, Hypothesis 3B addresses the impact of conspiracy theories on people’s behavior toward the same safety measures.

Using correlation analysis to evaluate the association between conspiracy theories and public health measures, we observed a moderate positive linear association between ConspTheory2 and PubHealthMes2 (*r*=0.66) and a moderate linear relationship between ConspTheory1 and PubHealthMes1 (*r*=0.53). The log-linear analysis showed that conspiracy theories are not significant predictors of PubHealthMes1 (*P*=.62) but are for PubHealthMes2 (*P*=.008). Thus, the null hypothesis that there is no association between conspiracy theories (ie, ConspTheory1) and people’s responses to public health (ie, wearing facial masks to limit the spread of COVID-19) is rejected. We can conclude that conspiracy theories are predictive of people’s behaviors in wearing facial masks.

We also analyzed the relationship between misinformation and behavior toward public health measures. Although the correlation and the predictive analyses showed a moderate positive linear relationship between misinformation and PubHealthMes1 (*r*=0.83), it is not predictive. The relationship between the two variables is just mathematical but not causal. Despite a negligible negative linear relationship between misinformation and wearing a facial mask (*r*=0.11), the log-linear model shows that misinformation is not a significant predictor for both PubHealthMes1 (*P*=.27) and PubHealthMes2 (*P*=.13). Notwithstanding the strong linear relationship between web searches to learn about misinformation and public health measures, there is no sufficient evidence to reject the null hypothesis. We can conclude that there is no association between learning about COVID-19 misinformation and people’s response to public health guidelines.

## Discussion

### Principal Findings

We found that people used search keywords related to past coronavirus epidemics (pastCoVepidemics) and the ongoing pandemic (presCoVpandemic) to learn about SARS-CoV-2 and COVID-19. However, the attention accorded to the pandemic led to less focus on terms relating to perennial illnesses (eg, “common cold,” “flu,” and more). These results corroborate studies reporting the unintended positive consequences of COVID-19 leading to declines in cases of influenza, flu, and similar infections (eg, Soo et al [46]). Other learning terms employed were keywords that addressed the pandemic directly. The average search indexes for those keywords were 19.01 for “nCoV” and “2019-nCoV,” 39.75 for “SARS-CoV-2,” and 32.40 for “COVID-19.”

Studies examining learning by web searching emphasized the significance of the search terms or phrases on what the users intended to learn. A trending word on the web indicates what information people are interested in learning [11,61,66]. This study identified the 25 most-used keywords to learn about SARS-CoV-2 and COVID-19 through web searches.

Regarding the impacts of what people learned on their behavior toward public health measures, the PCA identified three latent variables, classified as misinformation, ConspTheory1, and ConspTheory2. Only ConspTheory1 (“@5G”) directly and significantly influenced people’s behavior toward public health measures (ie, PubHealthMes2 [“wear mask”]). The conspiracy that 5G technology enhances the easy spread of COVID-19 [14] highlights danger, which can cause people to take precautions. A different study [15] identified erroneous beliefs in the 5G conspiracy theory as leading to the hoarding of essential goods during the initial period of the SAR-CoV-2 outbreak. Although there was a high correlation between misinformation (Tables 4 and 5) and behavior toward public health measures, this was not statistically significant based on the web search index. Also, as the pandemic lingers, thereby causing severe health and social crises, strains in family relations, and economic and business losses, many people are becoming increasingly aware of COVID-19 dangers [21,57,67-69]. Through direct impacts or by experience, this can cause changes in people’s behavior irrespective of whether they believed the misinformation or not.

### Strengths and Limitations

Internet platforms continue to play a significant role in health communication during the ongoing COVID-19 pandemic. Some studies attribute the increase in misinformation and conspiracy theories about COVID-19 in different countries to web searches, social media use, and online news media platforms that are used to learn about SARS-CoV-2 and COVID-19 [10,11,29]. However, most studies were anecdotal with no empirical evidence. Using Google Trends data, this study provides the first empirical evidence to this discourse. In the era of big data, the analysis of Google queries can be envisioned as a valuable tool for researchers to explore and predict human behavior, especially as studies suggest that online data can correlate with actual health data [70,71].

Infodemiology studies have their limitations too. While Google search keywords are short and easy to classify automatically, interpreting the terms semantically can be challenging. It is not clear why people are searching for these keywords. Furthermore, when using Google Trends, the sample is unknown and may not be representative, and individuals using the internet are not representative of the entire population. They are more likely to be younger, more educated, earn higher incomes, and reside in urban areas [18]. Individuals who are more likely to be severely affected by COVID-19 are not usually represented by this population [72,73]. Despite the identified limitations, previous studies suggest that web-based data provide valuable and valid results in exploring and predicting behavior and highly correlate with actual data [70,71]. Further, there are reports of rapid penetration of internet access and usage in different parts of the world, except for in regions with low internet penetration or countries with low scores in freedom of speech [22,74,75].

### Conclusions

The results of this empirical infodemiology study showed that a good portion of the global population learned about the outbreak of SARS-CoV-2 and COVID-19 through web searches, particularly in the early period of the pandemic. The period covers the initial days, weeks, and months from the emergence of the novel coronavirus in January 2020 up to June 30, 2020, when the public became more aware of the pandemic, especially after the first wave [1].

The PCA showed that people used the web to learn about the ongoing COVID-19 pandemic in two ways, namely, using pastCoVepidemics keywords and using presCoVpandemic keywords. The use of pastCoVepidemics keywords in web searches nosedived as the WHO formally named the novel coronavirus and the disease (ie, “nCoV,” “2019-nCoV,” “SARS-CoV-2,” and “COVID-19”) and, therefore, as these terms became available. The trends analysis showed that web searches used to learn about COVID-19 followed a similar trend as learning about public health measures, implying that the more that people focused their attention on learning about SARS-CoV-2 and COVID-19, the more they also learned about public health measures, and vice versa. Interestingly, learning about the conspiracy theory (ConspTheory1) that 5G technology contributes to the fast global spread of COVID-19 is a predictor of people’s behavior toward public health measures (PubHealthMes2). This erroneous belief makes people take precautionary measures, such as wearing a facial mask, although borne out of fear [14,15]. The same studies using the survey method also identified the same 5G-related conspiracy theory as making people respond out of fear to take precautions. This factor contributed to stockpiling of goods in the early days of the pandemic [15]. This study is the first to examine what people learned through web searches and how these influence people’s social behavior toward public health safety guidelines.

## Appendix

Multimedia Appendix 1 Summary statistics of the normalized daily global Google Trends scores for different keywords used in this study. Data correspond to the time window between January 1, 2020, and June 30, 2020 (N=182 data points).

## Abbreviations

2019-nCoV: 2019 novel coronavirus
CDC: Centers for Disease Control and Prevention
ConspTheory1: conspiracy theory 1
ConspTheory2: conspiracy theory 2
MERS: Middle East respiratory syndrome
pastCoVepidemics: keywords that address previous epidemics
PCA: principal component analysis
presCoVpandemic: keywords that explain the ongoing pandemic
PubHealthMes1: public health measures 1
PubHealthMes2: public health measures 2
Q-Q: quantile-quantile
SARS: severe acute respiratory syndrome
WHO: World Health Organization
