# Supplementary material for: Association Between What People Learned About COVID-19 Using Web Searches and Their Behavior Toward Public Health Guidelines: Empirical Infodemiology Study
Source: J Med Internet Res. 2021 Sep 2;23(9):e28975. doi: 10.2196/28975 (PMC8415385; doi:10.2196/28975)
Supplement: Multimedia Appendix 1 [file jmir_v23i9e28975_app1.docx]

**Multimedia Appendix 1. Google Trends Data Summary Statistics**

**Table S1:** Summary statistics of the normalized daily global Google Trends scores for different keywords used in this study. Data correspond to the time window between 1^st^ Jan 2020 to 30^th^ June 2020 (N=182)

|  | **Keywords** | **Min** | **Q1** | **Median** | **Mean** | **sd** | **Q3** | **Max** |
| --- | --- | --- | --- | --- | --- | --- | --- | --- |
| **COVID-19 & Related Epidemics** | 2019-nCoV | 0 | 3 | 7 | 15.62 | 22.43 | 16 | 100 |
|  | nCoV | 0 | 3 | 10 | 21.93 | 25.75 | 31.75 | 100 |
|  | SARS-CoV-2 | 0 | 5 | 42.5 | 39.75 | 29.59 | 64.75 | 100 |
|  | COVID-19 | 0 | 2 | 39 | 32.39 | 26.8 | 47 | 100 |
|  | Pandemic | 0 | 3 | 10.5 | 13.44 | 13.91 | 17 | 100 |
|  | MERS-CoV | 0 | 12 | 20.5 | 24.16 | 17.25 | 31.75 | 100 |
|  | MERS | 12 | 21 | 30 | 37.2 | 21.89 | 48.75 | 100 |
|  | SARS-CoV | 0 | 12 | 23.5 | 27.34 | 20.17 | 40.75 | 100 |
|  | SARS | 3 | 11 | 18.5 | 27.48 | 21.34 | 39.75 | 100 |
|  | Virus | 2 | 8.25 | 14 | 23.76 | 23.56 | 30.75 | 100 |
|  | Coronavirus | 0 | 11 | 16 | 27.13 | 25.97 | 38.75 | 100 |
|  | Influenza | 7 | 16 | 27 | 32.68 | 21.92 | 42 | 100 |
|  | FLU | 7 | 15 | 21 | 26.71 | 18.47 | 32 | 100 |
| **Misinformation** | Virus Hoax | 0 | 2 | 5 | 12.32 | 17.78 | 15 | 100 |
|  | Ingesting Bleach | 0 | 0 | 0 | 2.65 | 10.04 | 0 | 100 |
|  | “5G” technology enhancing the spread of the virus | 13 | 18 | 24 | 26.43 | 14.02 | 29 | 100 |
|  | COVID-19 Hoax | 0 | 0 | 11 | 16.5 | 23.3 | 25.5 | 100 |
|  | CommonCold2020 | 7 | 15 | 19 | 26.38 | 17.87 | 30 | 100 |
|  | China Virus | 0 | 2 | 9 | 14.59 | 18.22 | 18 | 100 |
|  | Bioweapons | 0 | 4 | 14.5 | 23.79 | 24.46 | 34 | 100 |
| **Public Health Measures** | Social Distancing | 0 | 0 | 18.5 | 22.85 | 24.45 | 33 | 100 |
|  | Hands Was | 15 | 23 | 29 | 33.97 | 18.46 | 34 | 100 |
|  | Wear a Facial Mask | 1 | 12 | 34 | 32.73 | 21.2 | 49 | 100 |
|  | Isolation | 9 | 14 | 18 | 26.1 | 20.72 | 28.75 | 100 |
|  | Quarantine | 0 | 4 | 17 | 22.25 | 21.45 | 32.75 | 100 |
